# Are Neutrophil Extracellular Traps Playing a Role in the Parasite Control in Active American Tegumentary Leishmaniasis Lesions?

**DOI:** 10.1371/journal.pone.0133063

**Published:** 2015-07-20

**Authors:** Fernanda Nazaré Morgado, Michelle T. C. Nascimento, Elvira M. Saraiva, Carla de Oliveira-Ribeiro, Maria de Fátima Madeira, Marcela da Costa-Santos, Erica C. F. Vasconcellos, Maria Ines F. Pimentel, Marcelo Rosandiski Lyra, Armando de Oliveira Schubach, Fátima Conceição-Silva

**Affiliations:** 1 Laboratório de Imunoparasitologia, Instituto Oswaldo Cruz IOC/FIOCRUZ, Rio de Janeiro, Brazil; 2 Departamento de Imunologia, Instituto de Microbiologia Paulo de Góes, Universidade Federal do Rio de Janeiro (UFRJ), Rio de Janeiro, Brazil; 3 Laboratório de Vigilância em Leishmanioses—VigiLeish, Instituto Nacional de Infectologia Evandro Chagas INI/FIOCRUZ, Rio de Janeiro, Brazil; The Hospital for Sick Children and The University of Toronto, CANADA

## Abstract

Neutrophil extracellular traps (NETs) have been described as a network of extracellular fibers composed by DNA, histones and various proteins/enzymes. Studies have demonstrated that NETs could be responsible for the trapping and elimination of a variety of infectious agents. In order to verify the presence of NETs in American tegumentary leishmaniasis (ATL) and their relationship with the presence of amastigotes we evaluated active cutaneous lesions of 35 patients before treatment by the detection of parasites, neutrophils (neutrophil elastase) and histones through immunohistochemistry and confocal immunofluorescence. Intact neutrophils could be detected in all ATL lesions. NETs were present in 27 patients (median 1.1; range from 0.1 to 23.5/mm^2^) with lesion duration ranging from one to seven months. NETs were in close proximity with neutrophils (r = 0.586; p = 0.0001) and amastigotes (r = 0.710; p = 0.0001). Two patterns of NET formation were detected: small homogeneously distributed networks observed in all lesions; and large structures that could be visualized at a lower magnification in lesions presenting at least 20% of neutrophils. Lesions presenting the larger NET formation showed high parasite detection. A correlation between NET size and the number of intact amastigotes was observed (p=0.02). As we detected an association between NET and amastigotes, our results suggest that neutrophil migration and NET formation could be stimulated and maintained by stimuli derived from the parasite burden/parasite antigen in the extracellular environment. The observation of areas containing only antigens not intermingled with NETs (elastase and histone) suggests that the involvement of these structures in the control of parasite burden is a dynamic process in which the formation of NETs is exhausted with the destruction of the parasites. Since NETs were also associated with granulomas, this trapping would favor the activity of macrophages in order to control the parasite burden.

## Introduction

Various infectious agents are able to invade and multiply inside human cells. Protozoa of the genus *Leishmania* are obligate intracellular parasites of the human mononuclear phagocyte system [1], but these parasites have also been detected inside neutrophils [2], fibroblasts [3,4], endothelial cells [5,6], and dendritic cells [7].

The function of macrophages as host and effector cells during infection with *Leishmania* parasites has been extensively studied. However, many questions regarding the ability of this protozoan to infect other cell types remain unanswered. In this aspect, controversy still exists whether neutrophils restrain or facilitate infection with *Leishmania sp* [8,9]. Neutrophils are the first cells recruited to the site of infection in leishmaniasis [10,11] and their participation in the control of parasite burden has been demonstrated in experimental models. Peters et al. [9] observed rapid and persistent infiltration of neutrophils at the site of inoculation of promastigotes by sandflies in a murine model within the first hours of infection with *L*. *major*. C57BL/6 and BALB/c mice depleted of neutrophils and infected with *L*. *major* showed higher parasite burdens, greater dissemination of infection and more severe lesions than non-depleted animals [8]. However, other investigators suggested a negative role of neutrophils in murine leishmaniasis, producing more severe disease associated with a Th2 response [12], or even serving as a carrier for *Leishmania* entry into macrophages [13]. Afonso et al [14] observed that human necrotic, but not apoptotic, neutrophils induced *in vitro* leishmanicidal activity mediated by macrophages. This leishmanicidal activity was dependent on TNF-α and neutrophil elastase (NE). Ribeiro-Gomes and Sacks [15] discussed the influence of early neutrophil-*Leishmania* interactions on the host immune response and suggested that infection outcome critically depends on the time of neutrophil recruitment and the tissue environment in which it occurs.

A particular form of death was described for neutrophils, which occurs with the release to the extracellular milieu of a mesh formed by chromatin associated with granular and cytoplasmic proteins named netosis from Neutrophil Extracellular Traps (NETs) [16]. NETs are endowed with the properties to arrest and kill different microorganisms [16–18]. During netosis, there is disruption of the nuclear membrane and chromatin decondensation, this last feature is dependent on the activity of elastase (NE), myeloperoxidase and peptidyl arginine deiminase 4 [18–20]. The formation of NETs has been reported in studies involving different pathogens such as *Mycobacterium tuberculosis* [21], fungi [22–23], HIV-1 [24], *Toxoplasma gondii* [25], *Eimeria bovis* [26], *Plasmodium falciparum* [27], *Leishmania amazonensis*, *L*. *major*, *L*. *chagasi* [28] and *L*. *donovani* [29]. Therefore, the in vitro interaction of neutrophils and *Leishmania* promastigotes [28, 29] leads to NET formation suggesting that NETs may contribute to the containment of promastigotes at the site of inoculation, thereby facilitating their uptake by mononuclear phagocytes [29]. NETs were also detected in the active lesion of a patient with American tegumentary leishmaniasis (ATL) [28]. However, the importance of this phenomenon in human tegumentary leishmaniasis lesions is still unknown, and it remains unclear whether NETs exert effects on the control of initial parasite burden *in vivo* or whether this effector mechanism occurs during an already established infection when only amastigote forms are present in lesions. It has been shown that amastigote forms of *L*. *amazonensis* induce NET release upon neutrophil interaction *in vitro* [28]. Therefore, in the present study we investigated the presence of NETs in active established ATL lesions and their relationship with *Leishmania* amastigotes.

## Materials and Methods

### Patients

Thirty-five patients with the localized cutaneous form of ATL were studied. Etiological diagnosis was confirmed by histopathological examination and isolation in culture as previously described [30]. Parasite characterization was possible in 11 isolates and *Leishmania (V*.*) braziliensis* infection was identified for all of them. All cases were from areas of Brazil (Rio de Janeiro) where *Leishmania (Viannia) braziliensis* is the infecting species of greatest prevalence. Patient age, lesion duration, number and site of the lesions were described (Table 1).

**Table 1:** Clinical and quantitative data of cutaneous lesions from 35 patients with American tegumentary leishmaniasis.

Patient number	Age (years)	Gender	Lesion Duration (month)	Number of lesions	Localization	MST^a^ (mm)	Neutrophil (%)	NET/mm^2^	Amastigotes/mm^2^
**1**	39	F	2.0	1.0	Lower limb	7.0	1.00	0.00	0.00
**2**	20	M	1.0	1.0	Lower Limb	ND^b^	13.54	1.30	11.00
**3**	45	M	2.0	1.0	Lower Limb	20.0	11.71	23.50	0.25
**4**	18	M	2.0	1.0	Lower limb	12.0	2.90	0.00	0.09
**5**	38	M	4.0	3.0	Upper limb	8.0	9.20	0.00	0.50
**6**	46	M	4.0	1.0	Upper limb	14.0	14.70	0.00	0.60
**7**	26	M	2.0	1.0	Upper limb	33.0	7.83	0.50	0.00
**8**	22	M	1.0	1.0	Face	ND^b^	10.33	0.00	0.00
**9**	59	F	3.0	1.0	Upper Limb	19.0	10.24	0.00	0.00
**10**	64	F	1.0	1.0	Neck	50.0	14.24	1.40	5.30
**11**	37	M	2.0	1.0	Upper Limb	15.0	11.22	0.85	4.00
**12**	33	F	1.0	1.0	Upper Limb	9.0	12.94	1.10	3.00
**13**	37	M	2.0	1.0	Lower Limb	2.0	11.00	0.10	0.00
**14**	14	F	2.0	1.0	Upper Limb	21.0	22.95	1.52	0.75
**15**	18	M	1.0	1.0	Lower Limb	23.0	7.04	0.21	0.06
**16**	39	M	3.0	2.0	Lower Limb	34.0	19.05	0.00	0.00
**17**	52	M	1.0	1.0	Upper limb	23.0	6.60	0.25	0.25
**18**	55	F	4.0	1.0	Upper Limb	5.0	15.82	0.00	0.50
**19**	40	F	3.0	2.0	Lower Limb	17.0	6.79	0.34	0.17
**20**	63	F	3.0	1.0	Trunk	49.0	18.33	0.36	0.18
**21**	43	F	1.0	1.0	Upper Limbs	ND^b^	17.84	10.30	30.00
**22**	23	M	2.0	1.0	Lower Limb	15.0	7.91	0.40	0.20
**23**	63	F	3.0	1.0	Trunk	49.0	22.03	10.50	40.00
**24**	27	F	1.0	1.0	Lower Limb	20.0	9.88	0.42	0.25
**25**	16	M	7.0	1.0	Neck	23.0	22.63	8.70	16.50
**26**	19	M	2.0	2.0	Trunk	12.0	16.15	0.45	0.23
**27**	36	M	5.0	1.0	Lower Limb	19.0	11.92	1.10	1.90
**28**	46	F	2.0	1.0	Head	38.0	9.15	0.67	0.34
**29**	41	F	2.0	2.0	Lower Limb	18.0	53.58	2.37	0.43
**30**	53	M	2.0	1.0	Lower limb	20.0	16.4	3.00	0.80
**31**	31	M	1.0	1.0	Upper limb	24.0	4.30	0.70	0.30
**32**	65	M	1.5	2.0	Upper limb	18.0	29.8	1.80	0.52
**33**	25	F	6.0	1.0	Upper limb	ND^b^	1.80	0.14	0.20
**34**	49	M	2.0	6.0	Trunk	14.0	34.8	14.60	0.90
**35**	69	F	1.5	1.0	Upper limb	23.0	37.7	19.30	13.6
**Median**	39	-	2.0	1.0	-	19.0	11.92	0.5	0.34
**Min-max**	14–69	-	1.0–7.0	1.0–6.0	-	2.0–50.0	1.0–53.6	0–23.5	0–40.0

^a^ MST—Montenegro skin test.

^b^ ND—not done.

### Ethics statement

The patients were included in the study after they had given formal written consent and the study was approved by the Ethics Committee of INI/FIOCRUZ (Comitê de Ética em Pesquisa do Instituto Nacional de Infectologia Evandro Chagas—CEP-INI—014/2001). When the patients were under 18 years old, informed consent was sign by parents or guardians, who also remained present throughout the clinical evaluation and diagnosis procedure.

### Immunohistochemistry (IHC)

Biopsy fragments were cut into 3-μm thick sections and mounted on silanized microscope slides (DakoCytomation, Carpinteria, CA, USA). The IHC was performed as described [31,32]. For single staining, the specimens were incubated with the primary antibodies anti-neutrophil elastase (NE- DakoCytomation, Carpinteria, USA) or anti-*L*. *braziliensis* polyclonal rabbit serum (Dr. Madeira-INI/FIOCRUZ), followed by sequential steps of washes in PBS (pH 7.4) and incubation with the biotinylated secondary antibody (Zymed, San Francisco, CA, USA), the streptavidin-biotin-peroxidase complex (ABC kit, DakoCytomation), and Aminoethyl carbazole (chromogen-AEC kit, Zymed). The slides were counterstained with Mayer’s hematoxylin (Dako). Double staining was performed for the colocalization of NE and amastigotes using the Dako Envision Doublestain System according to the manufacturers instructions. The slides were examined under a light microscope (Zeiss, Germany). The percentage of neutrophils was determined by counting 500 cells as standard and the number of amastigotes and NETs per mm^2^ tissue was calculated using a millimeter coverslip.

### Immunofluorescence and confocal microscopy

Biopsy fragments were obtained by cryosection and prepared for immunofluorescence microscopy as described [28]. Briefly, slides were rehydrated with PBS (pH 7.4) and unspecific epitopes were blocked with rabbit serum for two hours of incubation. Then, the sections were incubated with mouse anti-human NE (Calbiochem—EMD Millipore Co, Billerica, MA, USA)), anti-human DNA-histone complex (Millipore), anti-CD68 macrophages (Dako) and anti-*L*. *braziliensis* polyclonal rabbit serum, as primary antibodies. Anti-rabbit FITC-conjugated (Vector Labs), anti-mouse Alexa Fluor-546 (Calbiochem) and anti-mouse PE-conjugated were used as secondary antibodies. After staining, sections were mounted in Vectashield mounting medium (Vector Labs). Some slides were also mounted on medium containing DAPI (Fluoromount-G, eBioscience). Immunofluorescence analysis was done in a Nikon Eclipse E400 Microscopy (Nikon digital camera DXM1200F and Nikon ACT-1 software, Nikon, Japan). Confocal analysis were done in a Zeiss LSM 510 META confocal microscope (Program for Technological Development in Tools for Health—PDTIS-FIOCRUZ) using excitation at 488 nm or 546 nm and emission at 505–530 nm (band pass system) and 560 nm (long pass system). The images were processed using the program LSM Image Browser—Version 4.2.0.121 (Zeiss, Jena, Germany).

### Statistical analysis

The SPSS16 for Windows program (SPSS, Inc., Chicago, IL, USA) was used for statistical analysis. The results were analyzed using the nonparametric Mann-Whitney test and Spearman’s rank correlation coefficient for comparison between groups. The data are reported as the median and range. P-values ≤0.05 were considered as significant.

## Results

### Clinical data (Table 1)

Lesion biopsies from 15 female and 20 male patients located on the lower limbs (*n* = 13), upper limbs (*n* = 14), face (n = 2), and trunk (*n* = 6), with lesion duration ranged from 1 to 7 months were studied. Clinical characteristics are shown in Table 1.

### Neutrophils

**Fig 1:**
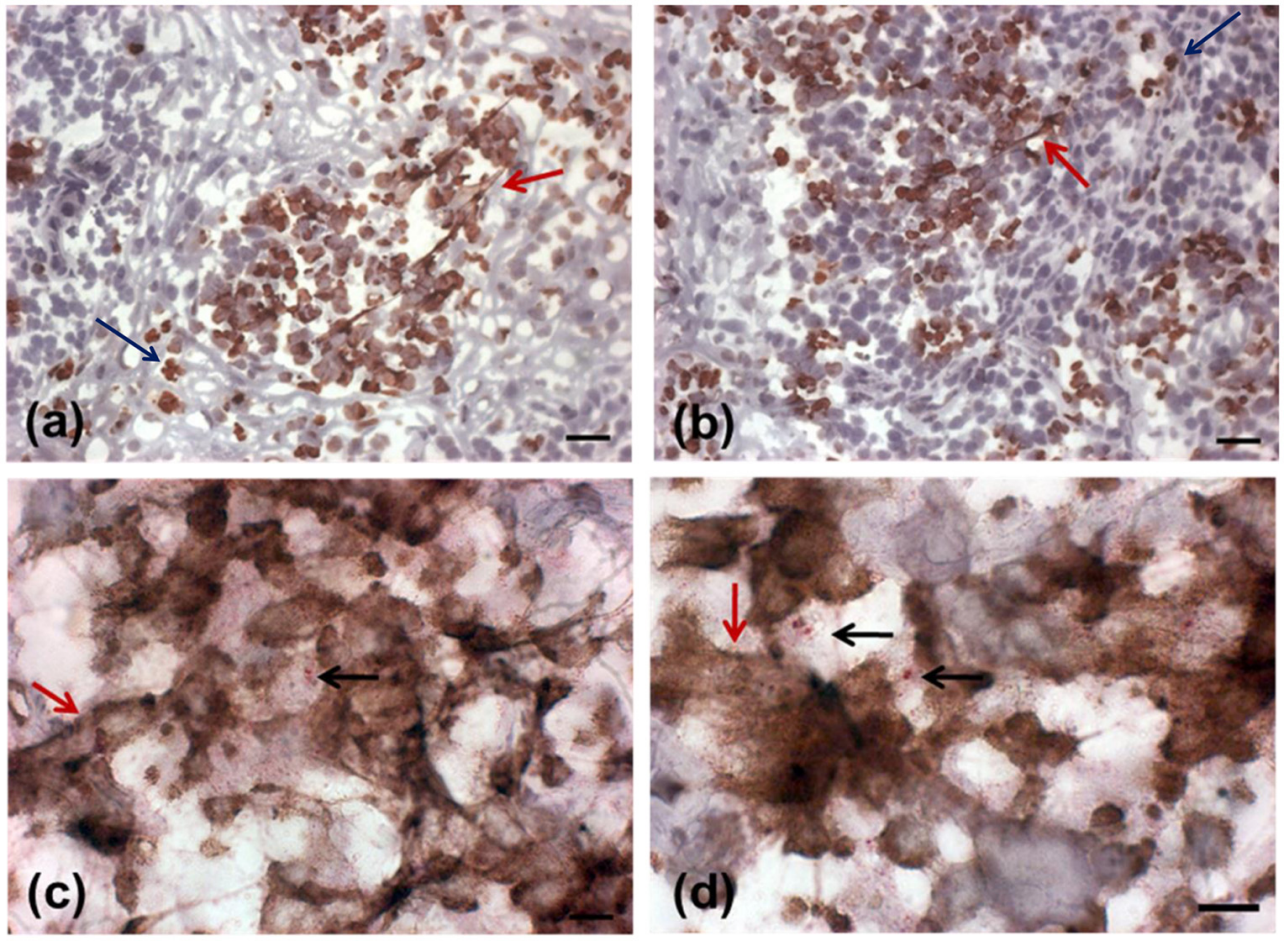
Detection of NETs in active lesions. (A, B) Presence of NETs in an active lesion detected by neutrophil elastase immunostain(red arrows) in sections counterstained with Mayer`s hematoxylin (bar = 25μm). (C, D) Colocalization of neutrophils, NETs and *Leishmania* amastigotes. (neutrophil elastase NETs: red arrows, neutrophils: blue arrows, amastigotes: black arrows) (bar = 10μm).

Neutrophils were heterogeneously distributed in the granulomatous inflammatory infiltrate in all lesions analyzed and were also present in clusters, inside vessels or adhered to the endothelium. The percentage of neutrophils ranged from 1.0 to 53.6% (median: 11.9%) (Table 1, Fig 1A and 1B, S1–S7 Figs).

### Neutrophil extracellular traps (NET)

NET formation was observed in 27 (77.1%) of 35 lesions analyzed. In positive cases, the distribution of NETs/mm^2^ ranged from 0.1 to 23.5 (median: 1.1) and the percentage of neutrophils ranged from 1.8 to 53.6% (median: 12.9%). In patients in whom no NETs were identified, the percentage of neutrophils ranged from 1.0 to 19.1% (median: 10.3%), without significant differences between the two groups. However, a correlation was observed between the number of NETs/tissue area and the percentage of neutrophils (r = 0.586; p = 0.0001), NETs and age (r = 0.368, p = 0.03), and NET and intensity of Montenegro skin test (MST) (r = 0.372; p = 0.039).

**Fig 2:**
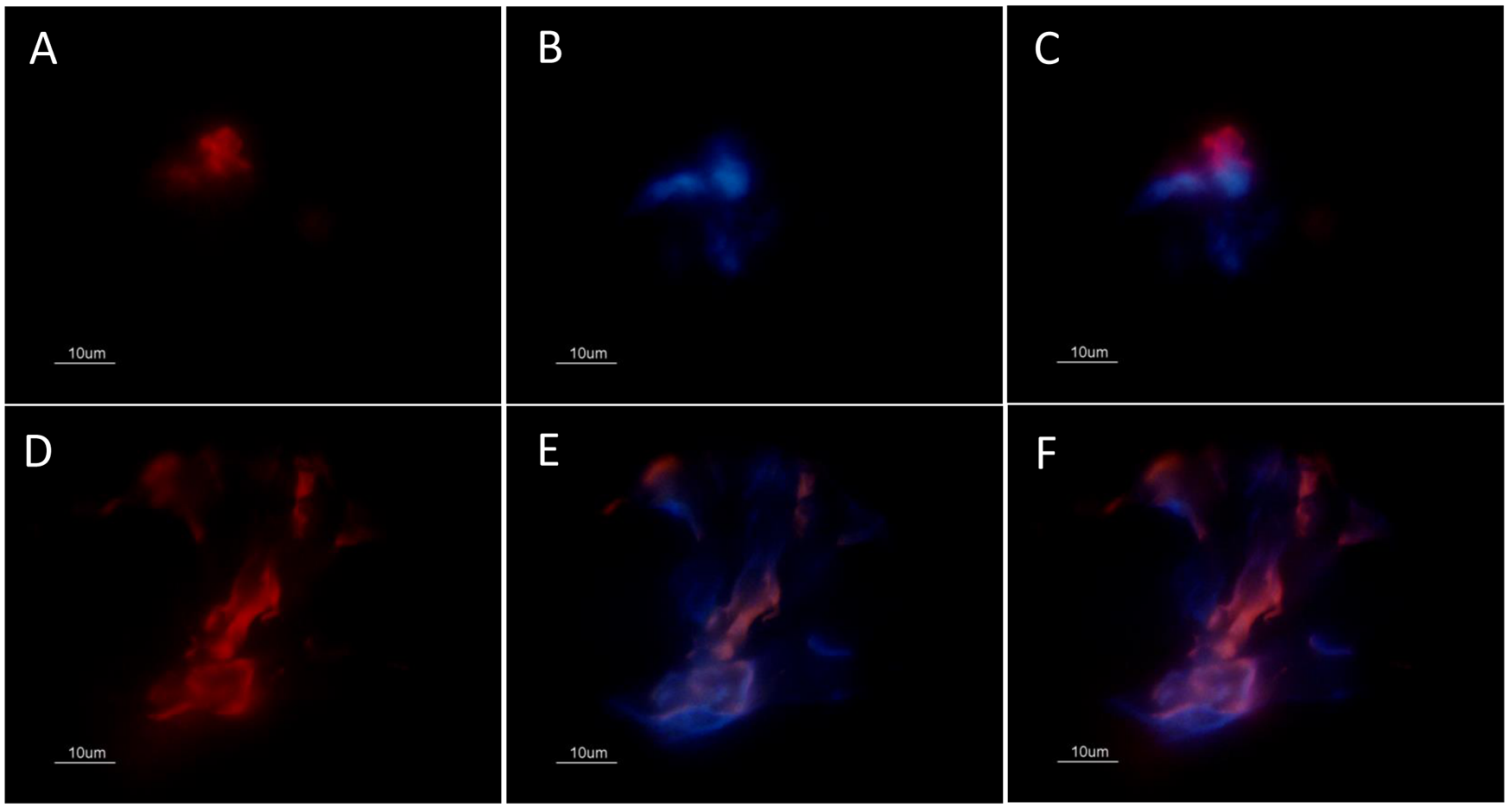
Colocalization analysis of neutrophil elastase and DNA by immunofluorescence. (A) lesion stained for neutrophil elastase (red), and (B) DNA (blue). (C) merge: elastase and DNA showing a neutrophil elastase positive cell with morphologically preserved nucleus. (D) NET containing neutrophil elastase, (E) and DNA. (F) merge: elastase and DNA. All images were obtained from different regions of the same lesion with 45 days duration before diagnosis. Bar = 10μm.

**Fig 3:**
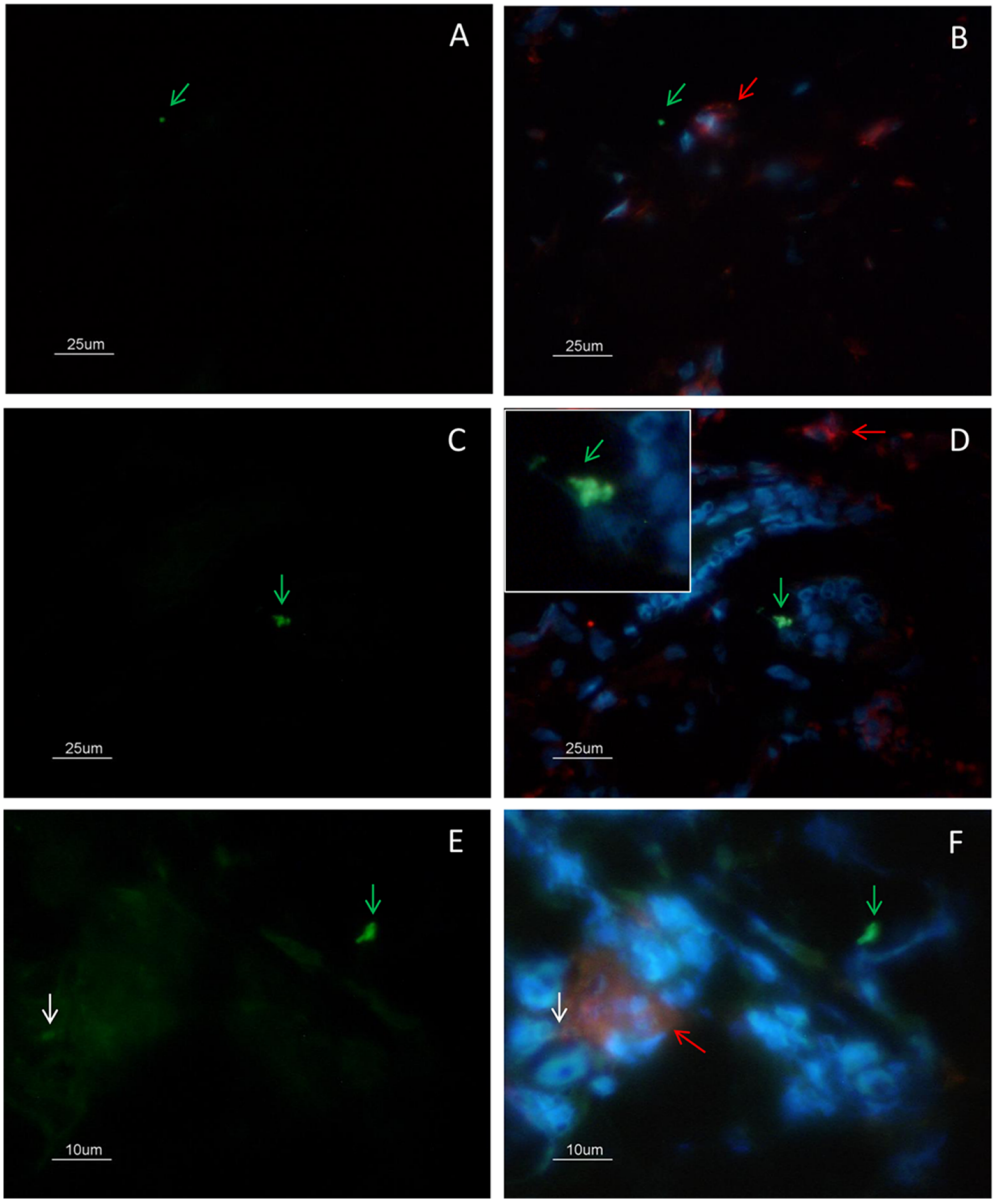
Colocalization analysis of CD68(+) macrophages and amastigotes by immunofluorescence. (A-B) Amastigotes (green arrow) were observed outside cells and close to macrophages (red arrow). (C-D) Amastigotes (green arrow) were observed inside CD68 negative cells (zoom). (E-F) Amastigote antigens inside macrophages (white arrow). (A-D) bar = 25μm; (E-F) bar = 10μm.

NETs were found throughout the lesion and varied in size according to the presence of neutrophils (Fig 1A–1D). Two patterns of NET formation were observed: (1) large heterogeneous webs consisting of large-diameter fibers that could be observed at low magnification (100 a 400x) identified when the percentage of neutrophils were higher than 20% (Fig 1A and 1B) and, (2) smaller homogeneous networks consisting of small-diameter fibers that could be better observed at 1000x magnification in all lesions presenting NET formation (Fig 1C and 1D). Fig 2 shows DNA and neutrophil elastase co-localization with typical features of NET formation (Fig 2D–2F), as well as neutrophil elastase positive cells presenting preserved nucleus (Fig 2A–2C). Since *Leishmania* amastigotes are typically found within lesion macrophages we evaluated the co-localization of macrophages and amastigotes. Amastigotes were observed outside cells (Fig 3A and 3B - green arrow) and inside CD68 negative cells (Fig 3C and 3D - zoom). We also observed amastigote antigens (white arrow) inside macrophages (Fig 3E and 3F - red arrow).

Since the presence of NETs was correlated with the number of neutrophils and since these cells might be involved in the control of parasite burden, we investigated the correlation between the presence of these structures and positivity for amastigotes and/or parasite antigens.

### Amastigotes and NETs

Amastigote forms were detected by immunohistochemistry in 29 (82.9%) of 35 patients studied (Table 1). From the six remaining patients four were also negative to NET and the other two presented NET formation with small diameters (0.10 and 0.50 NETs/mm^2^). Among the 29 lesions positive for amastigotes, 25 (86.2%) also presented NET scaffolds., In these patients, the number of amastigotes ranged from 0.06 to 40.0 / mm^2^ (median: 0.5) and the number of NETs ranged from 0.14 to 23.5 /mm^2^ (median: 1.1). A correlation was observed between the number of amastigotes/mm^2^ and the number of NETs/mm^2^ (r = 0.710; p = 0.0001). An association between the size of NETs and number of amastigotes could be observed (p = 0.02), since the lesions presenting larger NETs (n = 7; Table 1) showed the higher parasite burden (0.43 to 40.0 amastigotes/mm^2^ -Median 0.90) than the lesions presenting the small NETs (0 to 30.0 amastigotes/mm^2^ -Median 0.25). In the lesions of four patients who presented amastigotes in the absence of NETs, 0.09, 0.6, 0.5 and 0.5 amastigotes/mm^2^ were observed.

When the presence of amastigotes, neutrophils and NETs were compared, we noticed that all of them were in close proximity in various areas (Fig 1C and 1D, S8–S16 Figs). A correlation was observed between the number of amastigotes/mm^2^ and the percentage of neutrophils (r = 0.573; p = 0.0001). Amastigotes were often intermingled with NETs stained for elastase (Fig 1C and 1D). In addition, large clusters of NETs were noted close to sites containing a large number of parasites. In order to better demonstrate the correlation between the presence of NETs and amastigotes we performed a colocalization analysis by confocal microscopy.

**Fig 4:**
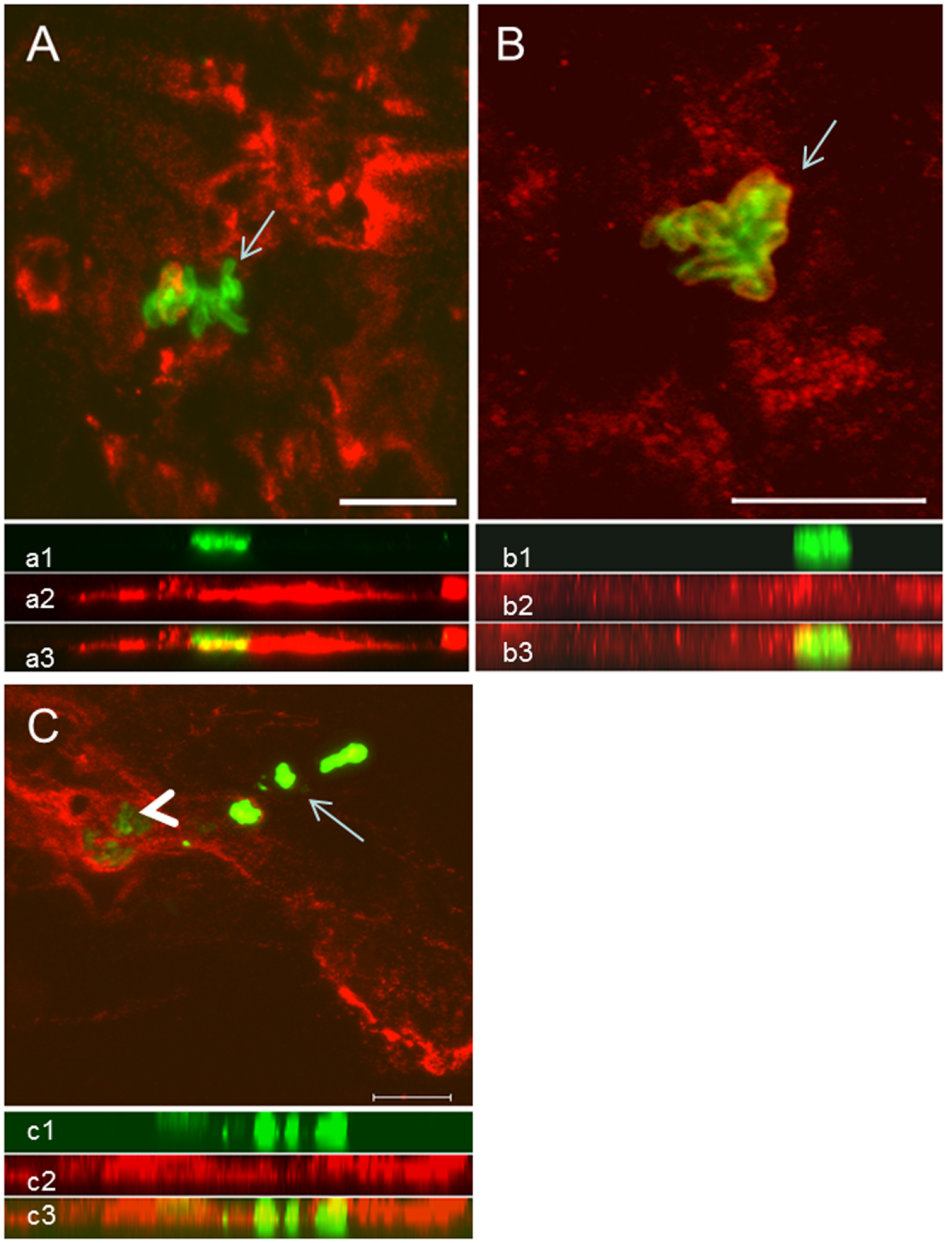
Colocalization analysis of NETs and amastigotes by confocal microscopy. (A) NETs containing neutrophil elastase (red) and amastigotes (green, arrow). (B) NETs containing histone (red) and amastigotes (green, arrow). (C) Area enclosing intact amastigotes (green—arrowhead) in intimate contact with NETs labeled with anti-neutrophil elastase (red). Area containing free *Leishmania sp* antigens (arrow). Below each image the respective side view (Z images) of (a1, b1, c1) amastigotes (green), (a2, c2) NET-elastase, (b2) NET-histones, merged sections showing the close contact between amastigotes and NETs (a3, b3, c3). Scale bar = 10μm.

The confocal images reinforced our immunohistochemical findings since extensive areas occupied by NETs, evidenced by NE and histone staining, are in close contact with nests plenty of amastigotes (Fig 4). NETs were scattered throughout the tissue, forming webs of different shapes and diameters. Colocalization analysis of the parasites and NETs showed that most intact amastigotes were surrounded by NETs (Fig 4A–4C, S17 and S18 Figs, S1 Video). On the other hand, areas positive for *Leishmania* antigens in which no intact parasites could be detected were arranged in a heterogeneous fashion and were not directly associated with the presence of NETs (Fig 4C, arrow), despite the presence of a surrounding inflammatory infiltrate and small numbers of neutrophils (data not shown).

## Discussion

In the present study, the characterization of NETs in the ATL lesions was done by evidencing some of its major components (DNA, NE and histones). Two patterns of NET formation were observed: 1) small homogeneously distributed webs, and 2) large structures that could be visualized at a lower magnification. Larger NETs were often identified where neutrophils were more concentrated forming clusters of cells. Brinkmann & Zychlinsky [33] described unfixed NETs as having a cloud-like appearance and occupying a space that is 10-15-fold bigger than the volume of the cells they originate from. In addition, we observed colocalization of amastigotes, neutrophils and NETs. When associated with NETs, amastigotes were intermingled with these webs, as also verified in an *in vitro* study of promastigote forms of *L*. *amazonensis* using electron and immunofluorescence microscopy [28]. In the present study, parasite burden was positively correlated with the number of neutrophils and with the number of NETs per tissue area. This finding suggests that in the late lesions the maintenance of neutrophil infiltration occurs in response to the presence of amastigotes in the extracellular environment. Moreover, the presence of NET and morphologically preserved neutrophils in association with amastigotes, regardless of the duration of the lesions, shows that this process might be related to a dynamic parasite clearance in the established infection. Our results suggested a dynamic process of NET formation when extracellular amastigotes are present. During cutaneous infection, amastigotes from *Leishmania* sp can be seen either inside or outside macrophages as we previously described [31]. The presence of neutrophils and/or NET would be important to clear extracellular amastigotes. Together with the macrophage activation and intracellular parasite destruction, the clearance of extracellular parasites by neutrophils would contribute to the parasite load control and, as a consequence, to the decrease of inflammatory process followed by lesion healing. The NET acumulation surrounding the extracellular amastigotesmay result in NET parasite trapping and, thus, impaired *Leishmania* dissemination, and also protozoan killing through close contact with NET toxic components. Corroborating this hypothesis, it was suggested that NETs may contribute to the containment of *L*. *donovani* promastigotes at the site of inoculation, thereby facilitating their uptake by mononuclear phagocytes [29]. Moreover, areas containing amorphous antigens of *Leishmania*, perhaps resulting from parasite killing were often not surrounded by NETs. The binomial NET/amastigotes could be induced by neutrophil chemotaxis which, in turn, is stimulated by extracellular amastigotes themselves and/or by IL-8 and *Leishmania* chemotactic factor [34,35], followed by stimulation of NET release. Finally, NET degradation would occur and macrophages could remove cell and antigen remnants. Here, we observed NETs associated to granulomatous inflammatory infiltrate in close proximity with amastigotes. Recently, Braian et al [36] analyzed human macrophages and NETs induced by *Mycobacterium tuberculosis*, and suggested that NETs can transfer danger signals (as that provided by Hsp72) to adjacent macrophages stimulating them to produce cytokines such as IL-6, TNF-α, IL-1β and IL-10. It has been shown that NETs activate NLRP3 inflammasome [37]. Moreover, extracellular trap formation has also been demonstrated for other types of granulocytes, such as eosinophils and mast cells [38,39], an even for macrophages [40,41]. However, as we have used specific markers for neutrophils, i.e. neutrophil elastase, colocalized with DNA, the events analyzed were due to the neutrophil extracellular traps.

Greater attention has been given over the last years to the function of neutrophils during subacute or chronic inflammatory processes. In the particular case of murine leishmaniasis, the results can vary. Lima et al [8] suggested the participation of neutrophils in the control of parasite burden since the absence of neutrophils resulted in the development of severe and disseminated disease. In contrast, Tacchini-Cottier et al [12] showed that the absence of neutrophils had a protective effect associated with a reduced Th2 response and partial resolution of lesions in susceptible BALB/c mice infected with *L*. *major*. This apparent contradiction might be due to differences in the mouse models and parasites used since Novais et al [42], studying BALB/c mice infected with *L*. *braziliensis*, observed that the elimination of neutrophils increased parasite multiplication. However, the co-inoculation of neutrophils with *L*. *braziliensis* had the opposite effect suggesting cooperation between neutrophils and macrophages associated to the production of TNF-α and superoxides. It has been well established that macrophage activation is insufficient in *L*. *major* infected BALB/c mice due to the production of IL-4 and the smaller neutrophil elastase production [43]. Furthermore, Zandbergen et al [13] showed that neutrophils may serve as vectors for the entry of *Leishmania* into macrophages, a model called Trojan horse. Neutrophils enter a process of apoptosis and, together with the parasite, are phagocytosed by macrophages, which became inactivated [2,13]. However, in the present study we evaluated the presence of extracellular regions of NE and histone suggesting NETosis that is independent of both apoptosis and necrosis [16,33]. In this context, using cells from resistant mice (C57Bl/6) infected with *L*. *major*, Ribeiro-Gomes et al [44] observed that the interaction between dead neutrophils and macrophages promoted the death of the parasite through the secretion of TNF-α. Fadok et al [45] demonstrated that NE was able to activate human macrophages and to induce the secretion of TNF-α. In addition, the inhibition of NE prevented the leishmanicidal activity of macrophages in a murine model, even in the presence of neutrophils, a fact suggesting the participation of NE in microbicidal activity [44]. Interestingly, during Gram-positive skin infection, Yipp et al [46] visualized live polymorphonuclear cells *in vivo* rapidly releasing NETs, which prevented systemic bacteria dissemination. NETosis occurred during crawling; thereby casting large areas of NETs associated with intact neutrophils that do not undergo lysis and contain phagocytosed pathogens retain the ability to multitask. In our study, active lesions of ATL also presented large areas of NETs composed NE and histones close to intact neutrophils distributed throughout the granulomatous infiltrate suggesting a probable cooperation between NETs, neutrophils and/or macrophages.

The duration of the lesions could play an important role in the relationship between neutrophil function and parasites. In this respect, during the first moment of the infection, *Leishmania* promastigotes may use the invasion of apoptotic neutrophils as an escape mechanism to establish infection in cells of the mononuclear phagocyte system [2,13]. Once a local inflammatory reaction is initiated, the proinflammatory environment would stimulate the degranulation and/or formation of NETs before the amastigote forms invade new cells, either neutrophils or macrophages. As a consequence, temporarily extracellular amastigotes would be destroyed, a process contributing to the reduction of local parasite burden. Our results show the presence of high numbers of neutrophils at the site of lesions and their intimate relationship with amastigotes, however it is important to mention that amastigotes could not be visualized inside neutrophils. In addition, all lesions studied already presented an important and diffusely distributed inflammatory process (data not shown and [31]).

Since their first description, the presence of NETs has been investigated in different infectious and non-infectious processes [16,47]. Within this context, three articles can be highlighted. In the first, NETs were identified in peripheral blood neutrophils of patients with malaria and associated with both severe malaria in children and mechanisms of protection in adults [27]. Knowledge about this relationship with disease severity might be important for studies investigating protection against this infection. Similarly, herein we observed positive correlations between NET formation and age, neutrophil percentage and age, as well as, NET and number of lesions. The second article, an *in vitro* study of the interaction between neutrophils and *L*. *amazonensis* promastigotes, demonstrated the trapping of these forms in the fiber networks [28]. In addition, the authors observed a toxic effect of histones released during the process of NET formation, reducing promastigote survival and observed extracellular regions of DNA and histone in human lesions suggesting NET activity during *in vivo* infection. In the third article, Bruns et al [22] showed that NETs are formed *in vivo* during the immune response against *Aspergillus fumigatus* and that their formation is a dynamic process. Although not playing a key role in the elimination of the fungus, the authors suggested that NETs exert a fungistatic effect, preventing dissemination of the fungus. In the same manner, NET could exert an additional parasiticidal effect, helping macrophages to clear parasites from the active lesions.

In conclusion, the present results suggest that 1) although neutrophils are classically identified as cells of the innate immune response, they can be detected throughout the inflammatory reaction observed in established ATL lesions; 2) the presence of NETs is directly related to the presence of intact amastigotes, irrespective of lesion duration, suggesting that their formation is maintained by the stimulus derived from the parasite burden/parasite antigen in the extracellular environment; 3) the observation of areas containing only antigens not intermingled with NETs (elastase and histone) suggests that the involvement of these structures in the control of parasite burden is a dynamic process in which the formation of these networks is exhausted with the destruction of the parasites; 4) the presence of extracellular parasite antigens could facilitate the activation of macrophages to the microbicidal stage, thus activating the control of parasite burden.

## Supporting Information

S1 Fig Neutrophil elastase and NET formation (brown). Inflammatory infiltrate in skin lesions of American tegumentary leishmaniasis. Counterstain—Meyer`s hematoxilin; 400x magnification.
(PDF)

S2 Fig Neutrophil elastase and NET formation (brown). Inflammatory infiltrate in skin lesions of American tegumentary leishmaniasis. Counterstain—Meyer`s hematoxilin; 400x magnification.
(PDF)

S3 Fig Neutrophil elastase and NET formation (brown). Inflammatory infiltrate in skin lesions of American tegumentary leishmaniasis. Counterstain—Meyer`s hematoxilin; 400x magnification.
(PDF)

S4 Fig Neutrophil elastase and NET formation (brown). Inflammatory infiltrate in skin lesions of American tegumentary leishmaniasis. Counterstain—Meyer`s hematoxilin; 400x magnification.
(PDF)

S5 Fig Neutrophil elastase and NET formation (brown). Inflammatory infiltrate in skin lesions of American tegumentary leishmaniasis. Counterstain—Meyer`s hematoxilin; 200x magnification.
(PDF)

S6 Fig Neutrophil elastase and NET formation (brown). Inflammatory infiltrate in skin lesions of American tegumentary leishmaniasis. Counterstain—Meyer`s hematoxilin; 200x magnification.
(PDF)

S7 Fig Neutrophil elastase and NET formation (brown). Inflammatory infiltrate in skin lesions of American tegumentary leishmaniasis. Counterstain—Meyer`s hematoxilin; 200x magnification.
(PDF)

S8 Fig Colocalization of neutrophil elastase (brown) and amastigotes (red). Inflammatory infiltrate in skin lesions of American tegumentary Leishmaniasis. counterstain—Meyer`s hematoxilin. 1000x magnification.
(PDF)

S9 Fig Colocalization of neutrophil elastase (brown) and amastigotes (red). Inflammatory infiltrate in skin lesions of American tegumentary Leishmaniasis. counterstain—Meyer`s hematoxilin. 1000x magnification.
(PDF)

S10 Fig Colocalization of neutrophil elastase (brown) and amastigotes (red). Inflammatory infiltrate in skin lesions of American tegumentary Leishmaniasis. counterstain—Meyer`s hematoxilin. 1000x magnification.
(PDF)

S11 Fig Colocalization of neutrophil elastase (brown) and amastigotes (red). Inflammatory infiltrate in skin lesions of American tegumentary Leishmaniasis. counterstain—Meyer`s hematoxilin. 1000x magnification.
(PDF)

S12 Fig Colocalization of neutrophil elastase (brown) and amastigotes (red). Inflammatory infiltrate in skin lesions of American tegumentary Leishmaniasis. counterstain—Meyer`s hematoxilin. 1000x magnification.
(PDF)

S13 Fig Colocalization of neutrophil elastase (brown) and amastigotes (red). Inflammatory infiltrate in skin lesions of American tegumentary Leishmaniasis. counterstain—Meyer`s hematoxilin. 1000x magnification.
(PDF)

S14 Fig Neutrophil elastase and NET formation (brown). Inflammatory infiltrate in skin lesions of American tegumentary Leishmaniasis. counterstain—Meyer`s hematoxilin. 1000x magnification.
(PDF)

S15 Fig Colocalization of neutrophil elastase (brown) and amastigotes (red). Inflammatory infiltrate in skin lesions of American tegumentary Leishmaniasis. counterstain—Meyer`s hematoxilin. 1000x magnification.
(PDF)

S16 Fig Colocalization of neutrophil elastase (brown) and amastigotes (red). Inflammatory infiltrate in skin lesions of American tegumentary Leishmaniasis. counterstain—Meyer`s hematoxilin. 1000x magnification.
(PDF)

S17 Fig Colocalization analysis by confocal microscopy. (Figure A) Neutrophil elastase (red) and amastigotes (green); (Figure B) histone (red) and amastigotes (green); (Figure C) histone (red) and degraded amastigotes (green). Scale bar = 10um.
(PDF)

S18 Fig Colocalization analysis by confocal microscopy. Neutrophil elastase (red) and amastigotes (green); Degraded amastigotes (arrow). Scale bar = 10um.
(PDF)

S1 Video Colocalization analysis by confocal microscopy. Area containing intact amastigotes (green) surrounded by NETs that contain neutrophil elastase (black arrow). Area containing free *Leishmania sp* antigens (white arrow). Scale bar = 10μm.
(AVI)
