# Supplementary material for: Are Neutrophil Extracellular Traps Playing a Role in the Parasite Control in Active American Tegumentary Leishmaniasis Lesions?
Source: PLoS One. 2015 Jul 20;10(7):e0133063. doi: 10.1371/journal.pone.0133063 (PMC4508047; doi:10.1371/journal.pone.0133063)
Supplement: S10 Fig — Inflammatory infiltrate in skin lesions of American tegumentary Leishmaniasis. counterstain—Meyer`s hematoxilin. 1000x magnification. (PDF) [file pone.0133063.s010.pdf]

Morgado et al

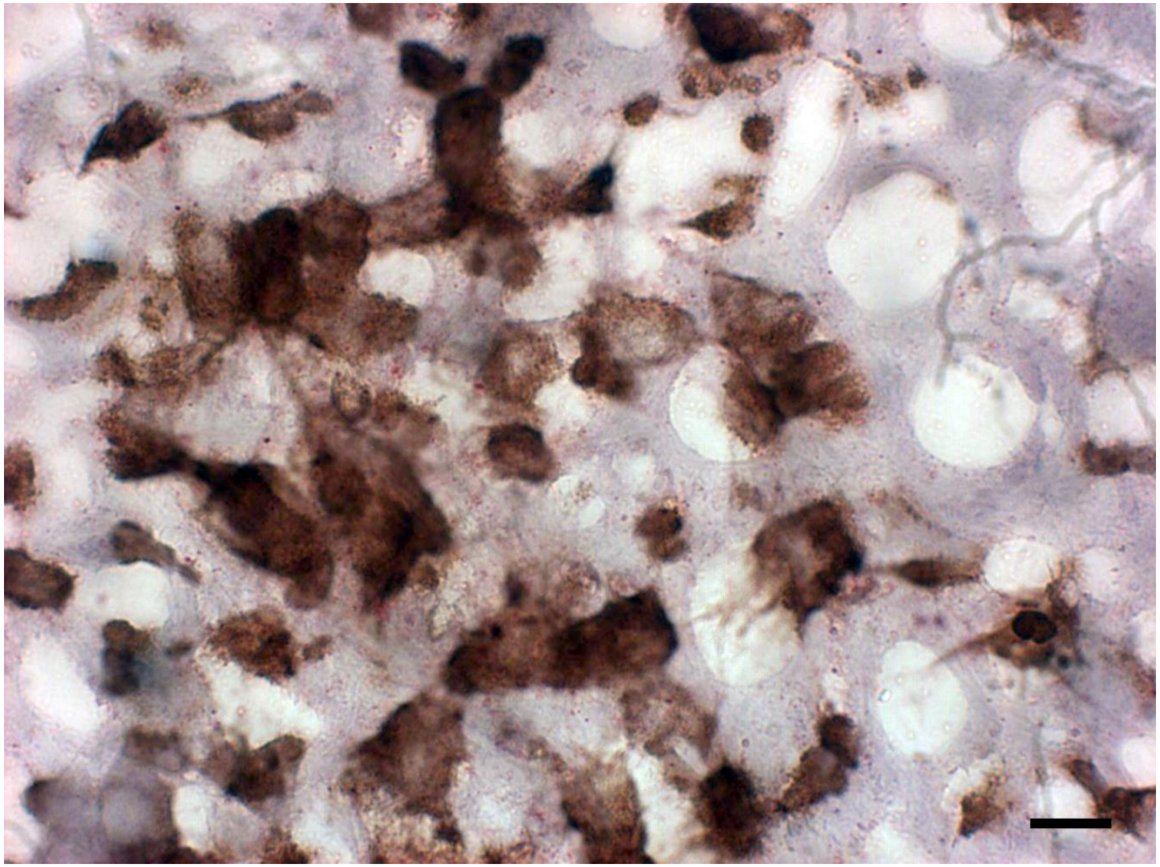

S10 Fig. Colocalization of neutrophil elastase (brown) and amastigotes (red). Inflammatory infiltrate in skin lesions of American tegumentary Leishmaniasis. counterstain – Meyer`s hematoxilin. 1000x magnification
