# Supplementary material for: Are Neutrophil Extracellular Traps Playing a Role in the Parasite Control in Active American Tegumentary Leishmaniasis Lesions?
Source: PLoS One. 2015 Jul 20;10(7):e0133063. doi: 10.1371/journal.pone.0133063 (PMC4508047; doi:10.1371/journal.pone.0133063)
Supplement: S17 Fig — (Figure A) Neutrophil elastase (red) and amastigotes (green); (Figure B) histone (red) and amastigotes (green); (Figure C) histone (red) and degraded amastigotes (green). Scale bar = 10um. (PDF) [file pone.0133063.s017.pdf]

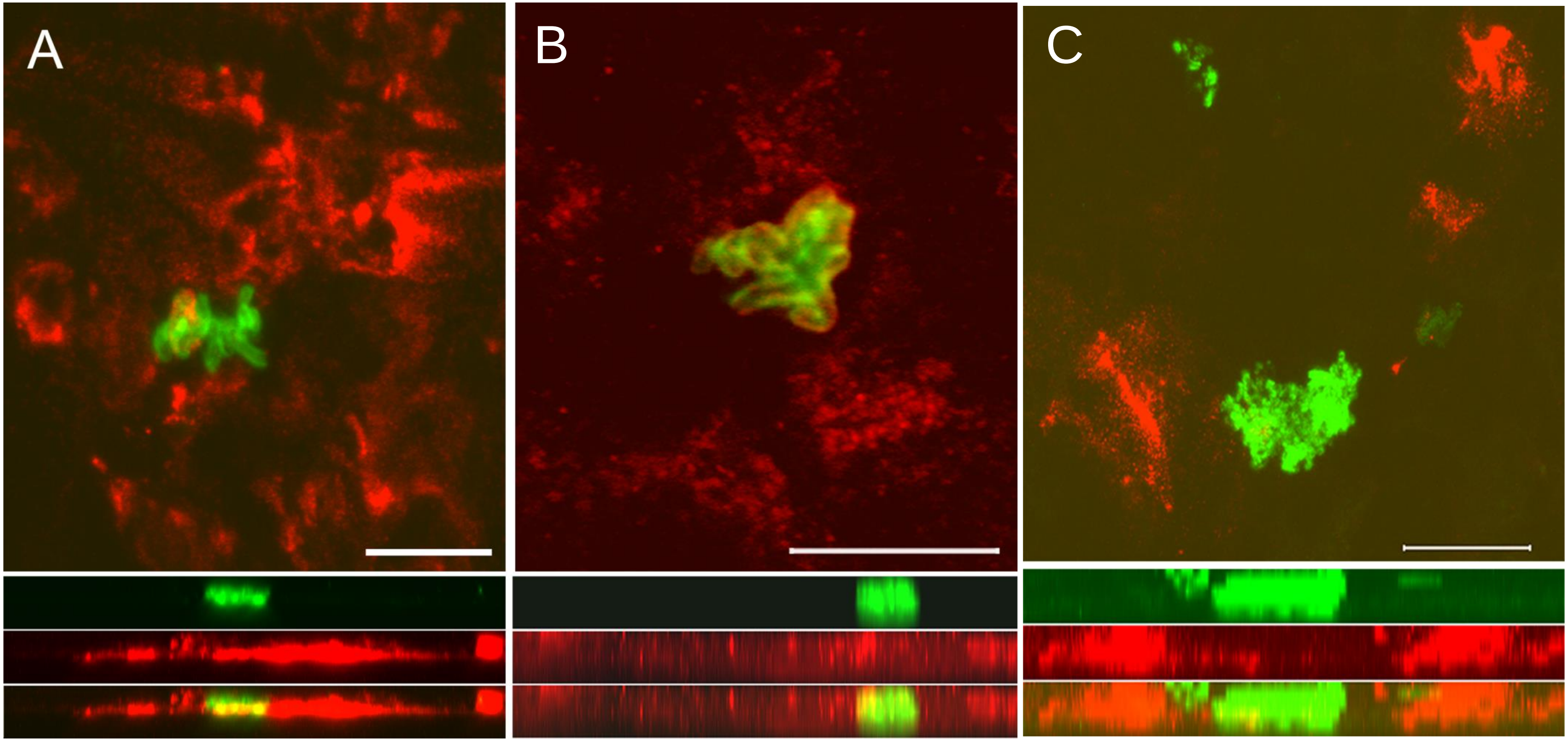

S17 Fig. Colocalization analysis by confocal microscopy. (Figure A) Neutrophil elastase (red) and amastigotes (green); (Figure B) histone (red) and amastigotes (green); (Figure C) histone (red) and degraded amastigotes (green). Scale bar = 10um

Morgado et al
