# Supplementary material for: Are Neutrophil Extracellular Traps Playing a Role in the Parasite Control in Active American Tegumentary Leishmaniasis Lesions?
Source: PLoS One. 2015 Jul 20;10(7):e0133063. doi: 10.1371/journal.pone.0133063 (PMC4508047; doi:10.1371/journal.pone.0133063)
Supplement: S18 Fig — Neutrophil elastase (red) and amastigotes (green); Degraded amastigotes (arrow). Scale bar = 10um. (PDF) [file pone.0133063.s018.pdf]

Morgado et al

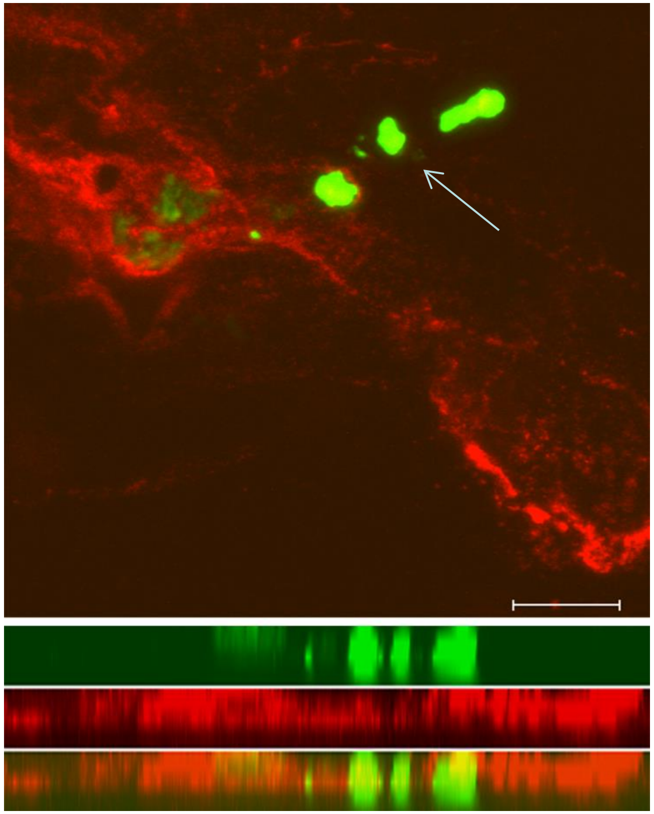

S18 Fig. Colocalization analysis by confocal microscopy. Neutrophil elastase (red) and amastigotes (green); Degraded amastigotes (arrow). Scale bar = 10um
